# The mechanism study of inhibition effect of prepared Radix Rehmanniainon combined with Radix Astragali osteoporosis through PI3K-AKT signaling pathway

**DOI:** 10.1590/acb371101

**Published:** 2023-01-06

**Authors:** Wen-qian Kang, Pei-feng Wei, Li Ou, Min Li, Chun-yu Liu

**Affiliations:** 1 MM. Shaanxi University of Chinese Medicine – Department of College of Pharmacy – Xianyang, China.; 2 MD. Shaanxi University of Chinese Medicine – Department of College of Pharmacy – Xianyang, China.

**Keywords:** Osteoporosis, Bone and Bones, Rats

## Abstract

**Purpose:**

To observe the mechanism of prepared Radix Rehmanniainon combined with Radix Astragali in treating osteoporosis.

**Methods:**

Osteoporosis rat model was established by bilateral ovariectomy combined with low-calcium diet feeding. Bone mineral density was measured by bone densitometer. Bone metabolism markers in serum were detected by enzyme linked immunosorbent assay (ELISA), bone tissue structure was observed by hematoxylin-eosin staining, and the effect of prepared Radix Rehmanniainon combined with Radix Astragali on PI3K-AKT signaling pathway was investigated by immunohistochemistry and reverse transcription polymerase chain reaction.

**Results:**

Compared with the model group, the bone tissue structure and imbalance of bone metabolism were improved, and the bone mineral density was significantly increased in the prepared Radix Rehmanniainon combined with Radix Astragali groups. After intervention with prepared Radix Rehmanniainon combined with Radix Astragali, the positive expression of PIK3CA and Akt1 in rat bone tissue was enhanced, and the expression levels of Akt1 mRNA were significantly increased.

**Conclusions:**

Prepared Radix Rehmanniainon combined with Radix Astragali may treat osteoporosis by activating PI3K/AKT pathway.

## Introduction

With the aging of the population in China, the incidence of osteoporosis is increasing year by year and has become an increasingly serious public health problem worldwide. Osteoporosis is a common skeletal disease closely related to age. It is mainly a systemic metabolic skeletal disease, prone to fracture due to bone mass loss, bone microstructure damage and increased bone fragility^1^. The causes of the disease are multiple factors, such as age, environment, living habits, and drugs. Osteoporosis is a serious threat to the bone health of middle-aged and elderly people, resulting in a sharp decline in the quality of life of middle-aged and elderly people, but also aggravated the social burden.

At present, western medicine treatment and preventive drugs for osteoporosis patients are mainly divided into two categories: bone resorption inhibitors (such as estrogen, isoflavones and calcium, etc.); and bone formation promoting agents (such as parathyroid hormone and steroids)^2^. However, these drugs have significant limitations in the treatment of osteoporosis, such as high price and significant side effects, and long-term use will make the body develop tolerance and affect its curative effect.

Traditional Chinese medicine (TCM) has a long history of treating osteoporosis, which has small toxic and side effects, moderate price, and it is one of the advantages of TCM syndrome differentiation^3^, which is worthy of clinical application. According to the theory of TCM, the etiology and pathogenesis of osteoporosis are related to deficiency of spleen and kidney^4^, and its pathogenesis is complex. Clinically, osteoporosisis mostly treated from the aspects of tonifying kidney and strengthening bone, tonifying kidney and filling essence and invigorating spleen and nourishing blood, and good results are obtained. Traditional medicine pays attention to drug compatibility in the treatment of diseases in order to adapt it to complex conditions, enhance drug efficacy and reduce drug toxic side effects. Clinical studies have found that the compatibility of *Rehmannia glutinosa* and *Astragalus membranaceus* can significantly increase bone mineral density, promote bone formation, and prevent and treat osteoporosis, and its utility is superior to that of single drugs^5^.

Prepared Radix Rehmannia (PRR) was first found in the Supplement to Materia Medica and has the functions of tonifying blood, nourishing yin, and benefiting essence filling the marrow. In recent years, it has been reported in a large number of literatures that PRR can promote bone formation, inhibit bone loss, and restore the dynamic balance between osteoblasts and osteoclasts, thus playing a role in anti-osteoporosis^6^^,^ 7. Radix Astragali (RA) is warm and sweet in nature and has the effects of invigorating the spleen and benefiting the lung, generating fluid and nourishing blood. RA decoction can reduce bone resorption and increase bone formation, thereby effectively preventing bone loss in ovariectomized rats^8^. Modern clinical pharmacological studies have shown that RA has various functions, such as protecting the kidney, preventing and treating osteoporosis, and resisting oxidative stress^9^. Our group found in previous studies that PRR-RA can increase bone mineral content, increase estrogen levels, improve bone metabolism, and increase bone mineral density in postmenopausal women^10^^,^ 11, but the molecular mechanism of its effect is not clear, so further in-depth study is still needed to guide the rational clinical application and bring more benefits to patients. PI3K-AKT signaling pathway is an important signaling pathway regulating cell proliferation, differentiation, and metabolic processes. In this study, enzyme linked immunosorbent assay (ELISA), immunohistochemistry, and reverse transcription polymerase chain reaction (RT-PCR) were used to further investigate the molecular mechanism of PRR-RA in the treatment of osteoporosis.

## Methods

### Experimental animals

Sixty specific-pathogen-free female Sprague Dawley rats were purchased from Chengdu Dashuo Laboratory Animal Co., Ltd., with body weight of 180 ± 20 g and certificate number of SCXK (Sichuan) 2020-030. The study was approved by the Ethics Committee of Shaanxi University of Traditional Chinese Medicine with ethical number of SUCMDL20220228003. The animal experiment was performed according to the guidelines for animal experiments of Shaanxi University of Traditional Chinese Medicine.

### Drugs and reagents

PRR (Bozhou Yonggang Herbal Pieces Factory Co., Ltd., batch number: 200630); RA (Guangdong Fengchun Pharmaceutical Co., Ltd., batch number: 200801A02); Alendronate sodium (Shiyao Group Ouyi Pharmaceutical Co., Ltd., batch number: 007200402, strength: 10 mg × 6t ablets); β-collagen degradation products (β-CTX); osteocalcin (BGP); bone alkaline phosphatase (BALP); tartrate resistant acid phosphatase 5b (TRAP5b); enzyme linked immunosorbent assay (ELISA) detection kit (Shanghai Enzyme-Linked Immunology Co., Ltd., batch number: ml004518, ml002883, ml004829 and ml029258); hematoxylin and eosin (HE) staining kit (Nanjing Jiancheng Technology Co., Ltd., batch number: 20200928); polymer anti-rabbit IgG-HRP kit, AKTI, PIK3CA (BOSTER, batch numbers: SV0002, A00024, PB0351); TRIzol™ Reagent Invitrogen™ (Invitrogen 15596018); RNase-Free Water (QIAGEN 129112); first strand cDNA synthesis kit (Invitrogen K1622); Quantity Nova SYBR green polymerase chain reaction (PCR) kit (QIAGEN 208052); tris-borate-EDTA (TBE) (Shanghai Weiao Biotechnology Co., Ltd.).

### Experimental instrument

Paraffin microtome S202 (Shenyang Yude Electronic Technology Co., Ltd.); ELX808 automatic microplate reader (Bio-Tek, United States of America); high-speed table-top centrifuge (Shanghai Anting Scientific Instrument Factory, model: TGL-16B); bone densitometer (Lunar, United States of America, model: Prodigy); tissue homogenizer (Beijing Dinghao Source Technology Co., Ltd., model: TL-2010S); fluorescence quantitative PCR instrument (Roche, model: Roche480II Real Time PCR System); incubator (Sanyo, Japan, model: MIR-262); and microscope (Olympus, Japan, model: BX51).

### Modeling and administration of experimental animals

After one week of adaptive feeding, the rats were randomly divided into PRR group, RA group, PRR-RA group, positive control group, model group, and control group. Ten animals per group. Except for the control group, rats in the other groups underwent bilateral oophorectomy and modeled in combination with a low-calcium diet. The rats were fasted and allowed free access to water 12 hours before surgery. After anesthesia with intraperitoneal injection of pentobarbital sodium, a longitudinal incision was made about 0.5-1 cm beside the midline of the back to find ovariectomy. Penicillin was injected intramuscularly for three days after surgery. Intragastric administration was performed on the seventh day after modeling. PRR group, RA group and PRR-RA group were given PRR decoction, RA decoction (5.4 g·kg^-1^), PRR-RA decoction (containing Astragalus 2.7 g·kg^-1^ and Rehmannia 2.7 g·kg^-1^, respectively). Positive group was given alendronate 1.05 mg·kg^-1^, and model group and control group were intragastrically administered with equal volume of pure water once a day for 12 weeks.

### Determination of bone mineral density

Twelve hours after the last intragastric administration, the rats were anesthetized by intraperitoneal injection of pentobarbital sodium. The parameters of lumbar spine and left tibia were analyzed by bone densitometer.

### Collection of samples

The rats were anesthetized, blood was collected and killed, and the required samples were collected. Samples included: whole blood, left tibia, and lumbar vertebrae of all groups of rats. The blood was centrifuged at 3,000 R/min for 15 min, and the upper serum was taken and stored in the -80 °C refrigerator for standby. The left tibia and lumbar spine were fixed in 4% paraformaldehyde.

### Serum bone metabolism markers

After blood collection from abdominal aorta, serum was collected by centrifugation for the detection of serum bone metabolism markers β-CTX, BGP, BALP, and TRAP5b. The detection method was determined according to the ELISA kit instructions. The absorbance at 450 nm with automatic microplate reader was detected, the value was record, and then the content of marker was converted according to the standard curve.

### Observation of bone tissue structure in rats

The tibiae and lumbar vertebrae of rats were fixed with 4% paraformaldehyde and decalcified with 10% EDTA. Specimens were embedded and cut into 5-μm-thick sections. After staining with HE staining solution, it was observed under a microscope.

### The positive expression levels of PIK3CA and Akt1 in bone tissues were detected by immunohistochemistry

Sections were deparaffinized to water by xylene I, xylene II and graded ethanol. Endogenous peroxidase was inactivated by soaking in 3% H_2_O_2_ at room temperature for 30 min. Distilled water 2 min × 3 times; serum blocking solution was dropped at room temperature for 30 min; primary antibody at a dilution of 1:300 was dropped overnight at 4 °C, and 0.01M phosphate buffered saline (PBS) was used instead of primary antibody as a negative control. PBS was washed 5 min × 3 times; secondary antibody was added dropwise and 37 °C for 30 min. PBS was washed 5 min × 4 times; diaminobenzidine (DAB) was developed, counterstained with hematoxylin, and washed thoroughly with water; dehydrated, transparent, mounted and photographed. The results were analyzed with ImageJ software.

### RT-PCR to detect the expression of PIK3CA and Akt1 in bone tissues

Total RNA was extracted from bone tissue using Trizol one-step method, and cDNA was obtained after reverse transcription. β-actin was set as an internal reference gene, and PIK3CA and Akt1 were used as target genes. Fluorescent quantitative PCR primers for the genes are listed in Table 1. PCR reaction system: 2 × SYBGEEN PCR mix 5 μL, primer 1 μL, cDNA template 2.5 μL, H_2_O 1.5 μL. Reaction conditions: 95 °C for 2 min; 95 °C for 5 s, 60 °C for 10 s, 45 cycles. Each sample was tested in triplicate. The relative gene expression was calculated by 2^-ΔΔCT^.

**Table 1:** Primer sequences for real-time fluorescence quantitative polymerase chain reaction.

Amplification length	Primer name	Sequence (5’-3’)
64 bp	β-actin forward	Ctaaggccaaccgtgaaaag
	β-actin reverse	tacatggctggggtgttga
86 bp	PIK3CA forward	ttcctgatcttcctcgtgct
	PIK3CA reverse	cggacagtgctcctccttag
176 bp	Akt1 forward	actcattccagacccacgac
	Akt1 reverse	ccggtacaccacgttcttct

### Data analysis

Tests were performed using GraphPad Prism 7 software. Measured data are presented as mean ± standard deviation (x ± s). For data conforming to normal distribution, one-way analysis of variance (ANOVA) was used for statistical evaluation for comparison among multiple groups, and rank sum test was used if normal distribution was not followed. *P* < 0.05 was considered statistically significant.

## Results

### Effects on bone turnover markers

Compared with the control group, the levels of bone formation markers BGP and BALP in the model group were significantly reduced, β-CTX and TRACP-5b increased (*P* < 0.01). Compared with the model group, the levels of BGP and BALP of rats in each group increased after treatment. The levels of CTX and TRAP5b decreased, and the levels of BGP and BALP in the PRR-RA group increased significantly (*P* < 0.01). The level of CTX decreased significantly (*P* < 0.01), and the level of TRAP5b decreased, but the difference was not statistically significant (Table 2).

**Table 2:** Changes of BGP, BALP, β-CTX and TRAP5b in rat serum#.

Group	BGP (ng/mL)	BALP (ng/mL)	β-CTX (ng/mL)	TRACP-5b (ng/mL)
Control	6.65 ± 2.04	4.66 ± 1.15	14.06 ± 2.62	1.59 ± 0.42
Model	3.51 ± 1.03##	2.59 ± 0.83##	35.87 ± 6.86##	2.44 ± 0.58##
Alendronate sodium	5.79 ± 1.08**	3.83 ± 1.02**	21.23 ± 3.95**	2.30 ± 0.56
PRR	5.45 ± 0.95**	3.37 ± 0.73	28.73 ± 5.42*	2.04 ± 0.53
RA	5.03 ± 1.38*	3.51 ± 0.77*	26.86 ± 2.13**	2.13 ± 0.62
PRR + RA	6.25 ± 1.24**	4.12 ± 1.28**	23.19 ± 3.76**	2.17 ± 0.38

BGP: osteocalcin; BALP: bone alkaline phosphatase; β-CTX: β-collagen degradation products; TRAP5b: tartrate resistant acid phosphatase 5b; PRR: prepared Radix Rehmannia; RA: Radix Astragali;

# data are presented as mean ± standard deviation of 10 rats in each group: compared with the control group,

## *P* < 0.01; compared with the model group,

* *P* < 0.05;

** *P* < 0.01.

### Effects on bone tissue structure in rats

**Figure 1:**
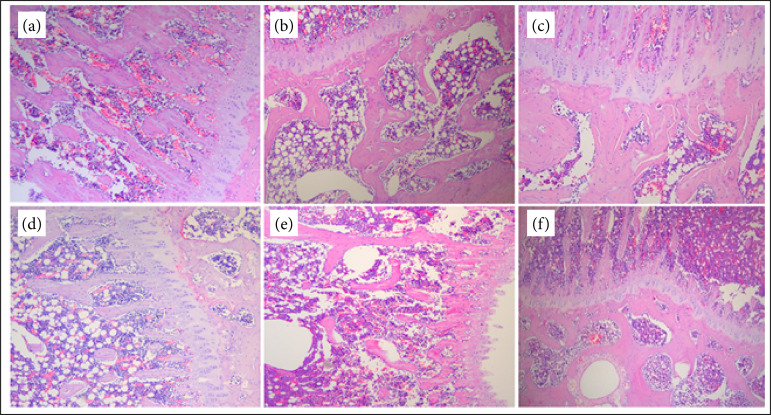
Hematoxilin and eosin (HE) staining of rat tibia (HE ×40). **(a)** Control group; **(b)** model group; **(c)** Alendronate sodium group; **(d)** PRR group; **(e)** RA group; **(f)** PRR + RA group.

PRR: prepared Radix Rehmannia; RA: Radix Astragali.

In the control group, the shape of tibial trabecular bone was intact and mesh-like, osteocytes were neatly and evenly arranged, and bone microstructure was intact. Compared with the blank group, the tibial reticular formation of rats in the model group was destroyed, osteocytes were disorganized, and the intramedullary space was enlarged, indicating the occurrence of osteoporosis, suggesting that the model was successfully established. Compared with the model group, the number of bone beams in the bone marrow region of model rats in each treatment group increased, a few were unevenly arranged, and the bone tissue structure was significantly improved (Fig. 1).

### Effects on bone mineral density in rats

**Figure 2:**
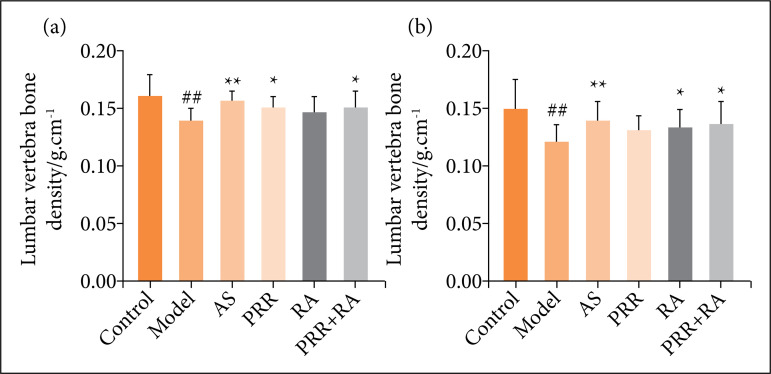
Comparison of bone mineral density in various groups of rats after 12 weeks of drug intervention^#^.

PRR: prepared Radix Rehmannia; RA: Radix Astragali; AS: alendronate sodium; ^#^compared with the control group, ^##^ *P* < 0.01; compared with the model group, *P < 0.05, **P < 0.01.

As shown in Fig. 2, compared with the control group, the bone mineral density of the left tibia and lumbar vertebra of rats in the model group was significantly decreased (*P* < 0.01). Compared with the model group, the bone mineral density of rats in the PRR, RA, PRR-RA, and Alendronate sodium groups were increased.

### Effect on positive expression levels of PIK3CA and Akt1 in rat bone tissue

**Figure 3:**
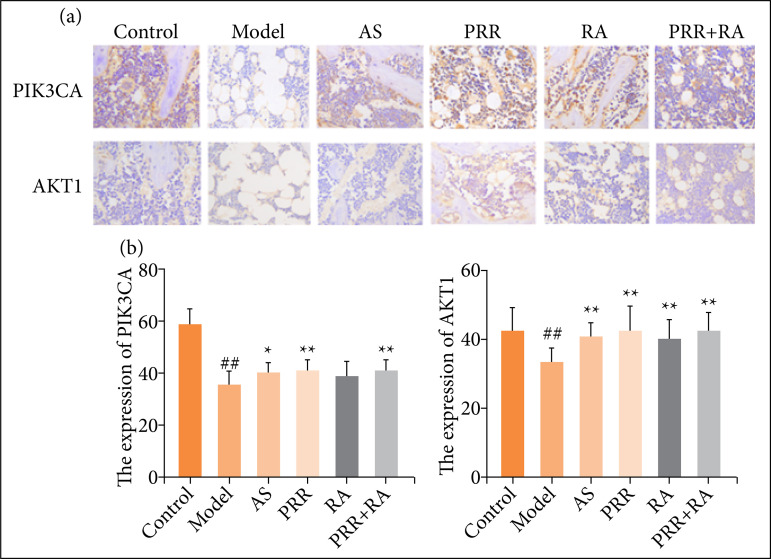
**(a)** Expression levels of PIK3CA and Akt1 in rat bone tissue (× 200); **(b)** changes of PIK3CA and Akt1 expression levels in rat bone tissue^#^.

PRR: prepared Radix Rehmannia; RA: Radix Astragali; AS: alendronate sodium; ^#^compared with the control group, ^##^ *P* < 0.01; compared with the model group, *P < 0.05, **P < 0.01.

The immunohistochemical staining results (Fig. 3) showed that the positive expression levels of PIK3CA and Akt1 in bone tissues of the model group were significantly decreased compared with those of the control group (*P* < 0.01). Compared with the model group, the positive expression levels of PIK3CA and Akt1 in bone tissues of rats in each group increased after administration, in which the positive expression levels of PIK3CA and Akt1 in the bone tissues of the PRR-RA group were significantly increased (*P* < 0.01).

### Effects on PIK3CA and Akt1 mRNA expression levels in rat bone tissue

**Figure 4:**
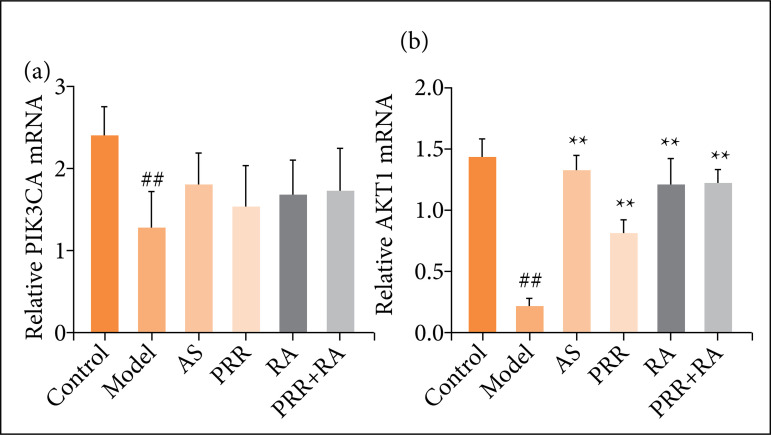
Comparison of PIK3CA and Akt1 expressionin bone tissue of rats in each group^#^.

PRR: prepared Radix Rehmannia; RA: Radix Astragali; AS: alendronate sodium; ^#^compared with the control group, ^##^ *P* < 0.01; compared with the model group, *P < 0.05, **P < 0.01.

According to the results of RT-PCR, the values obtained were calculated according to the 2^-ΔΔCT^ method, and it could be seen from the results of Fig. 4 that the expression levels of PIK3CA and Akt1 mRNA in the bone tissues of the model group were significantly decreased compared with those of the blank group (*P* < 0.01). After treatment, the expression of PIK3CA and Akt1 mRNA in bone tissue of rats in each group increased to different extents, and the expression of Akt1 mRNA in bone tissue of PRR-RA group was significantly enhanced (*P* < 0.01), and the expression of PIK3CA increased, but there was no statistically significant difference (Fig. 4).

## Discussion

Osteoporosis occurs as a result of multiple factors, multiple links, and multiple goals. It has a long course and provokes great harm, which seriously affects the physical health of middle-aged and elderly people. Globally, osteoporosis causes more than 8.9 million fractures each year. According to statistics, the probability of osteoporosis in the elderly over 60 years of age in China is 36%, including 23% in men and 49% in women, which indicates that osteoporosis has become a major public health problem faced nowadays^12^.Therefore, it is of great significance to study and improve the pathogenesis and drug mechanism of osteoporosis for its prevention and treatment , and it is also the focus of attention all over the world.

The model of bilateral ovariectomized rats is thought to closely resemble osteoporosis in postmenopausal women. The success rate of modeling in this model is not only high, but also simple and easy to operate, the mortality rate of rats is low, and it can better simulate the process of osteoporosis development. It is a recognized and recommended modeling method for osteoporosis at home and abroad^13^.

Studies have shown^14^ that, after ovariectomy in rats, bone strength and bone mineral density decreased, and bone resorption was greater than bone formation, and this change was just consistent with osteoporosis in postmenopausal women. Bone mineral density is the gold standard for the diagnosis of osteoporosis^15^. Therefore, the success of rat modeling can be judged by measuring bone mineral density. In this study, bilateral ovariectomy combined with low-calcium diet feeding was used to establish osteoporosis model, which could rapidly reduce bone mineral density and show osteoporosis in osteoporosis animal model. By detecting the bone mineral density of left tibia and lumbar vertebra in each group, the results showed that the bone mineral density of left tibia and lumbar vertebra in the model group was significantly lower than that in the blank group (*P* < 0.01), indicating that the osteoporosis model was successfully reproduced by removing bilateral ovaries in this experiment.

Recent studies have shown that biochemical markers of bone turnover are biochemical products reflecting skeletal cell activity and bone matrix metabolism levels and have become important indicators for the diagnosis of metabolic bone disease and prediction of fracture risk^16^. BALP and BGP are specific biochemical markers reflecting bone formation^17^. BGP mainly regulates and maintains bone calcium, which can reflect the osteogenic activity of the body to a certain extent. If the activity of osteoblasts is enhanced, the serum BGP level in the body is increased; conversely, if the osteoclastic activity is enhanced, the serum BGP level in the body will decrease, followed by osteoporosis.

BALP is a glycoprotein secreted by osteoblasts, which promotes the production of bone tissue, and can reflect the activity of bone remodeling, and its expression is closely related to bone development^18^. Serum β-CTX is a kind of bone metabolism index with high sensitivity originating from bone matrix or osteocytes, which can reflect the bone metabolism in time. Increased levels of these markers indicate risk of developing osteoporosis. TRACP-5b is considered a good marker for understanding bone resorption and osteoclast activity^19^, and its index is significantly increased if osteoporosis symptoms occur.

In this experiment, the levels of bone formation markers BGP and BALP in the model group were significantly decreased, and β-CTX and TRACP-5b were increased (*P* < 0.01), indicating that the rats developed osteoporosissymptoms, suggesting that the model was successfully established. After treatment, the levels of BGP and BALP in PRR-RA group were significantly increased (*P* < 0.01), the level of β-CTX was significantly decreased (*P* < 0.01), and the level of TRAP5b was also decreased to some extent. The experimental results suggest that PRR-RA can improve the imbalance of bone resorption and bone formation in osteoporosis and can also have a positive effect in regulating bone metabolism.

PI3K/AKT signaling plays a crucial role in the regulation of bone formation and bone resorption. It is involved in the inhibitory effect of osteoporosis by promoting osteoblast proliferation, differentiation, and bone formation, which in turn controls bone mineral density balance^20^. In addition, the PI3K/Akt pathway can also influence osteoclast formation. Mice lacking Akt1 have been found to show insufficient bone mass gain at an early age and osteopenia at adulthood, reflecting the potential osteoblast-specific effects of the PI3K/Akt signaling pathway^21^. In contrast, activation of Akt increased bone mass by blocking phosphatidylinositol-3,4,5-triphosphate 3-phosphatase expression in mouse osteoblasts.

It has been shown that up-regulation of PI3K/Akt pathway can activate and prevent the development of inflammation, promote bone mineralization, improve bone biomechanics, and reduce bone tissue injury in postmenopausal osteoporosis rats^22^. It inhibits osteoblast apoptosis in osteoporotic mice by regulating the PI3K/Akt signaling pathway^23^. The results of PCR and immunohistochemical staining showed that PRR-RA could significantly increase the expression levels of PIK3CA and Akt in PI3K/Akt1 signaling pathway after intervention, indicating that PRR-RA could promote the process of bone formation by activating PI3K/Akt signaling pathway to maintain the balance of bone metabolism.

## Conclusion

The mechanism of PRR-RA in improving osteoporosis may be related to activation of PI3K-AKT signaling pathway.
