# ERRATA

**DOI:** 10.1590/acb371101.errata

**Published:** 2023-03-13

In the manuscript **The mechanism study of inhibition effect of prepared Radix Rehmanniainon combined with Radix Astragali osteoporosis through PI3K-AKT signaling pathway**, which DOI is: https://doi.org/10.1590/acb371101, published in the journal **Acta Cirúrgica Brasileira**, 2022;37(11)e371101, page 5, in the axis Y of Figure 2(b):

**Instead of**: lumbar vertebra bone

**Should be**: left tibia bone

